# Magnesium Sulfate-Induced Ophthalmoplegia: A Rare Postnatal Complication

**DOI:** 10.7759/cureus.85918

**Published:** 2025-06-13

**Authors:** Pranit Khandait, Himali Hatwar

**Affiliations:** 1 Neurology, Midas Multispeciality Hospital, Nagpur, IND; 2 Obstetrics and Gynecology, Government Medical College and Hospital, Nagpur, Nagpur, IND

**Keywords:** hypermagnesemia, magnesium sulfate toxicity, mgso₄, mgso₄-induced neurotoxicity, ophthalmoplegia

## Abstract

Magnesium sulfate (MgSO_4_) is widely used in managing eclampsia, but its neurological side effects are often overlooked. A 25-year-old woman with a second gravida pregnancy developed eclampsia in her third trimester and underwent an emergency cesarean section. In the immediate postoperative period, she presented with bilateral ophthalmoplegia, ptosis, and neck flexion weakness, though her pupillary reflexes remained intact. Initial concerns included myasthenia gravis and Miller-Fisher syndrome, but further investigations, including repetitive nerve stimulation (RNS), acetylcholine receptor (AChR), and muscle-specific kinase (MuSK) antibody testing, were negative. Three Tesla MRI brains with constructive interference in steady state (CISS) sequences of III, IV, and VI were normal, and laboratory results revealed elevated serum magnesium levels (5.5 meq/L). A diagnosis of hypermagnesemia-induced ophthalmoplegia was confirmed, and MgSO_4_ therapy was promptly discontinued. Within 36 hours, her symptoms began to resolve, highlighting the transient yet significant neuromuscular effects of magnesium toxicity.

This case underscores the importance of recognizing early signs of magnesium toxicity in postpartum patients receiving MgSO_4_ therapy. While mild hypermagnesemia can cause headache and diminished reflexes, severe cases may progress to muscle paralysis, respiratory failure, and even cardiac arrest. Ophthalmoplegia, though rare, should raise suspicion, prompting timely magnesium level monitoring and treatment adjustments. Withholding MgSO_4_ at the appropriate time can prevent further complications, ensuring safer management of eclampsia and postpartum neurological health.

## Introduction

Magnesium sulfate (MgSO_4_) is a widely used anticonvulsant in the management of preeclampsia and eclampsia due to its neuroprotective and vasodilatory properties. It acts as a calcium antagonist, reducing neuronal excitability and preventing seizures. However, its toxic effects, particularly at supra-therapeutic levels, can result in significant neuromuscular and cardiac complications. While respiratory depression and hypotension are well-documented, rare neurological manifestations such as ophthalmoplegia often go unrecognized. This report presents a rare case of MgSO_4_-induced bilateral ophthalmoplegia in a postpartum patient, shedding light on its pathogenesis and emphasizing the importance of early detection and management.

## Case presentation

A 25-year-old female, gravida 2, at 36 weeks of gestation, presented with a one-week history of bilateral pedal edema, accompanied by a persistent, diffuse headache and intermittent episodes of visual blurring. These symptoms gradually intensified until, one day prior to admission, she experienced a generalized tonic-clonic seizure. At that time, her blood pressure was 164/90 mmHg, and proteinuria was present. This clinical deterioration led to a diagnosis of eclampsia.

In accordance with standard obstetric protocols, she was immediately started on an intravenous MgSO_4_ regimen following the Pritchard protocol - a regimen designed to achieve therapeutic magnesium levels that prevent recurrent seizures. Given the maternal risks and the need for rapid stabilization, an emergency lower-segment cesarean section was performed, and the renal function test was within normal limits (Table 1).

**Table 1:** Laboratory investigation SGPT: serum glutamic pyruvic transaminase; ALT: alanine aminotransferase; SGOT: serum glutamic oxaloacetic transaminase; AST: aspartate aminotransferase; AChR: acetylcholine receptor; MuSK: muscle-specific kinase; CSF: cerebrospinal fluid

Parameter	Result	Reference range	Interpretation
Serum magnesium	5.5 mEq/L	1.5-2.5 mEq/L	Elevated
Serum creatinine	0.7 mg/dL	0.6-1.2 mg/dL	Normal
Blood urea	11 mg/dL	7-20 mg/dL	Normal
Total bilirubin	0.9 mg/dL	0.2-1.2 mg/dL	Normal
Conjugated bilirubin	0.3 mg/dL	0.0-0.3 mg/dL	Normal
SGPT (ALT)	18 U/L	7-56 U/L	Normal
SGOT (AST)	15 U/L	10-40 U/L	Normal
Total protein	7.5 g/dL	6.4-8.3 g/dL	Normal
Albumin	3.9 g/dL	3.5-5.0 g/dL	Normal
AChR antibody	<0.05 nmol/L	<0.25 nmol/L (negative)	Negative
MuSK antibody	<0.05 nmol/L	<0.05 nmol/L (negative)	Negative
CSF glucose	55 mg/dL	45-80 mg/dL (or 60-70% of blood glucose)	Normal
Corresponding blood glucose	90 mg/dL	70-110 mg/dL	Normal
CSF protein	42 mg/dL	15-45 mg/dL	Normal
CSF cell count	2 cells/µL	0-5 cells/µL	Normal

In the immediate postoperative period, the patient developed notable neurological symptoms. She had bilateral complete ptosis and ophthalmoplegia, characterized by a total restriction of eye movements. Her pupils remained reactive to light, indicating that parasympathetic innervation was largely spared. Additionally, she experienced mild weakness in neck flexion, absent knee-deep tendon reflexes, and a completely normal sensory examination.

**Figure 1:**
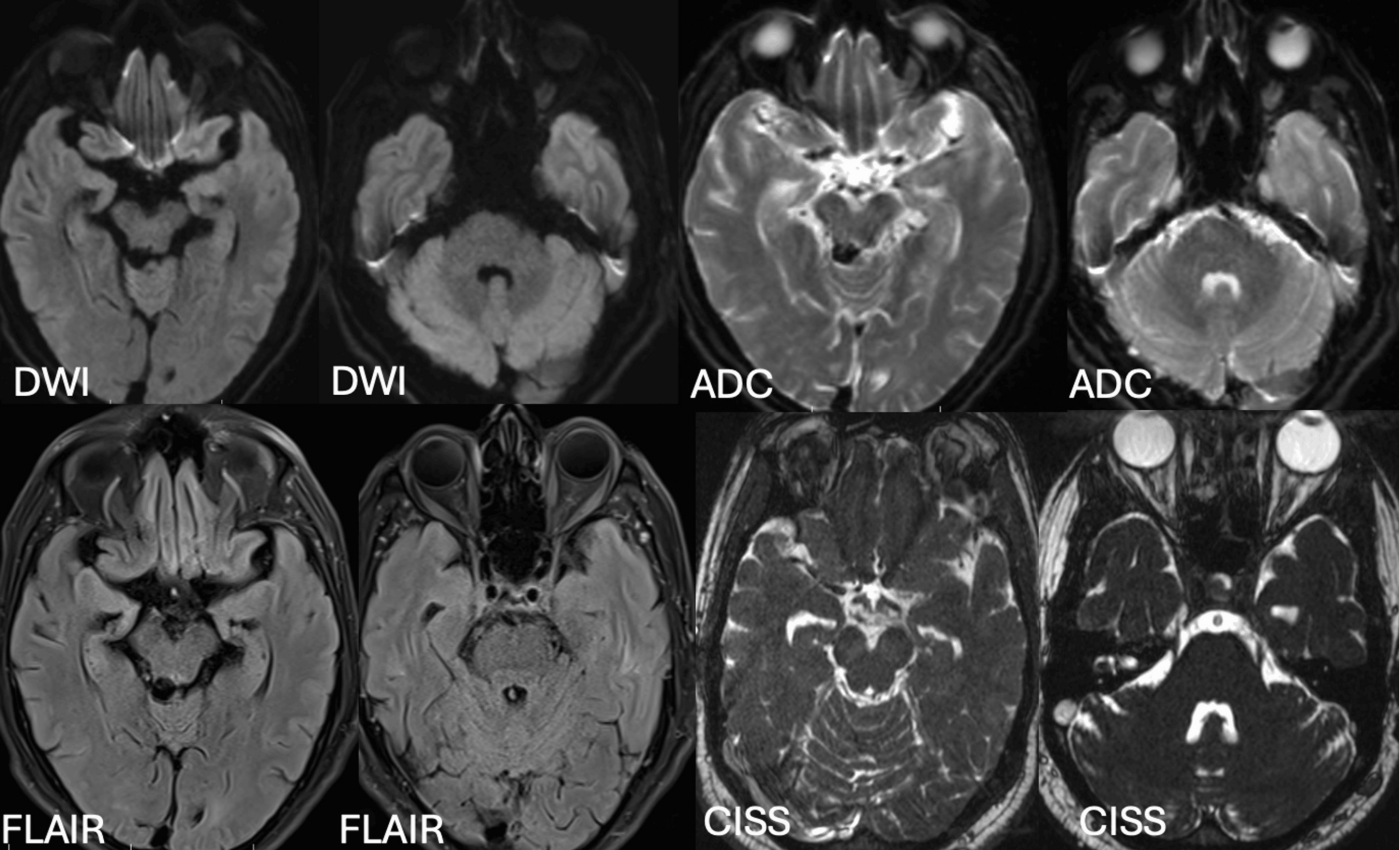
MRI brain plain and CISS sequence within normal limits DWI: diffusion-weighted imaging; ADC: apparent diffusion coefficient; FLAIR: fluid-attenuated inversion recovery; CISS: constructive interference in steady state

Given the acute presentation of these neuromuscular deficits, the differential diagnosis initially included conditions such as myasthenia gravis, Miller-Fisher syndrome (a variant of Guillain-Barré syndrome), and brainstem stroke. However, extensive investigations, including brain MRI with constructive interference in steady state (CISS) sequence for cranial nerves III, IV, and VI, were normal (Figure 1).

Repetitive nerve stimulation (RNS) at 3 Hz, conducted on the bilateral orbicularis oculi, bilateral nasalis, right-sided deltoid, anconeus, and abductor pollicis brevis (APB) muscles, was within normal limits. The absence of a decremental response on RNS testing, along with negative anti-acetylcholine receptor (anti-AChR) and anti-muscle-specific kinase (MuSK) antibody assays, effectively ruled out myasthenia gravis.

The above finding confirmed the diagnosis of hypermagnesemia-induced neuromuscular dysfunction, as described in Table 1. The excessive magnesium likely exerted its effects by competitively inhibiting calcium at presynaptic sites of the neuromuscular junction, thereby reducing acetylcholine release and leading to the observed neuromuscular blockade - a phenomenon that, while beneficial in preventing seizures, can result in adverse effects when magnesium accumulates. Recognizing the risk, MgSO_4_ therapy was immediately discontinued, and supportive care measures were instituted, including calcium gluconate.

Following these interventions, the patient’s clinical condition improved significantly. Within 24 hours, a noticeable improvement in her neuromuscular function was evident, and by 48 hours, both the ophthalmoplegia and ptosis had completely resolved as serum magnesium levels normalized to 2.5 mEq/L. The resolution of symptoms following the cessation of MgSO_4_ therapy confirmed the diagnosis of hypermagnesemia-induced ophthalmoplegia and underscored the reversibility of this condition when promptly managed.

The management of hypermagnesemia involves several critical steps. First, discontinuation of the MgSO_4_ infusion is essential to halt further accumulation. Intravenous hydration is then administered to enhance the renal excretion of magnesium. In cases where serum magnesium levels are dangerously high or significant neuromuscular blockade is present, calcium gluconate is used as an antidote to counteract the neuromuscular effects of magnesium. Serial monitoring of serum magnesium levels is vital to ensure they remain within the therapeutic range and to prevent further iatrogenic toxicity.

This case highlights not only the efficacy of MgSO_4_ in preventing eclamptic seizures but also the potential for serious, albeit reversible, neuromuscular complications when serum magnesium levels exceed the optimal range. It emphasizes the importance of careful patient monitoring, particularly in the postpartum period, and serves as a reminder of the delicate balance clinicians must maintain between therapeutic benefits and adverse effects in the management of eclampsia.

## Discussion

MgSO_4_ is a mainstay in the management of preeclampsia and eclampsia due to its anticonvulsant properties, which are mediated by its action as a calcium channel antagonist. At therapeutic doses, MgSO_4_ helps to stabilize neuronal membranes by reducing calcium-mediated acetylcholine release at the neuromuscular junction [1]. However, when serum magnesium levels exceed the therapeutic range, patients may develop signs of neuromuscular blockade such as decreased deep tendon reflexes, generalized muscle weakness, and, in rare instances, cranial nerve dysfunction leading to ophthalmoplegia [2]. In the case presented, the patient’s serum magnesium level reached 5.0 meq/L, which induced bilateral ophthalmoplegia - an uncommon manifestation where the extraocular muscles are selectively affected, while pupillary responses remain intact. This differential sensitivity may be due to inherent variations in the neuromuscular junctions of extraocular versus other skeletal muscles [3].

The pathophysiology of magnesium toxicity is rooted in its ability to inhibit presynaptic calcium influx, thereby reducing neurotransmitter (acetylcholine) release at the neuromuscular junction [4]. Experimental studies have demonstrated that elevated magnesium levels can significantly impair neuromuscular transmission, accounting for the clinical presentation of muscle weakness and, in severe cases, respiratory depression. For serum magnesium levels of 4-6 mEq/L, patients may experience headache, drowsiness, and diminished deep tendon reflexes. At levels of 6-10 mEq/L, symptoms can progress to somnolence, hypocalcemia, absent deep tendon reflexes, hypotension, bradycardia, and ECG changes. When serum magnesium exceeds 10 mEq/L, muscle paralysis may occur, leading to flaccid quadriplegia, apnea and respiratory failure, complete heart block, and cardiac arrest [5]. Although hypermagnesemia typically presents with generalized neuromuscular symptoms, isolated cranial nerve involvement - such as ophthalmoplegia - has been documented in a few case reports, suggesting that certain muscle groups may be more vulnerable to magnesium’s effects [6]. Differential diagnoses in such cases include myasthenia gravis, Miller-Fisher syndrome, and brainstem stroke; however, the absence of decremental responses on RNS, normal imaging, and negative antibody testing help to rule out these conditions [7,8].

Current guidelines emphasize the importance of serial monitoring of serum magnesium levels during MgSO_4_ therapy, especially in patients with risk factors like renal impairment, to prevent toxicity [9]. Management of magnesium toxicity primarily involves the prompt discontinuation of MgSO_4_, supportive care such as intravenous hydration, and, in severe cases, the administration of calcium gluconate to antagonize magnesium’s neuromuscular effects [10]. Early recognition and intervention are critical, as illustrated in this case, where the timely cessation of MgSO_4_ led to rapid clinical improvement and complete resolution of symptoms within 48 hours. This report, therefore, underscores the need for heightened clinical vigilance and strict adherence to monitoring protocols during MgSO_4_ administration to balance its therapeutic benefits against the risk of toxicity, even in patients with no identifiable risk factors for MgSO_4_ toxicity.

## Conclusions

Hypermagnesemia-induced ophthalmoplegia, though rare, should be recognized as a potential complication of IV MgSO_4_ therapy in postpartum patients. Timely identification, discontinuation of MgSO_4_, and supportive management lead to rapid symptom resolution. Routine serum magnesium monitoring is crucial to balance the benefits of MgSO_4_ therapy with the risk of toxicity. Clinicians should maintain a high index of suspicion for magnesium-induced neuromuscular dysfunction in patients presenting with unexplained cranial nerve deficits.
